# Heart rate variability and risk of agitation in Alzheimer’s disease: the Atherosclerosis Risk in Communities Study

**DOI:** 10.1093/braincomms/fcad269

**Published:** 2023-10-13

**Authors:** Kathy Y Liu, Eric A Whitsel, Gerardo Heiss, Priya Palta, Suzanne Reeves, Feng V Lin, Mara Mather, Jonathan P Roiser, Robert Howard

**Affiliations:** Division of Psychiatry, University College London, London W1T 7NF, UK; Department of Epidemiology, Gillings School of Global Public Health, University of North Carolina at Chapel Hill, Chapel Hill, NC 27599, USA; Department of Medicine, School of Medicine, University of North Carolina at Chapel Hill, Chapel Hill, NC 27599, USA; Department of Epidemiology, Gillings School of Global Public Health, University of North Carolina at Chapel Hill, Chapel Hill, NC 27599, USA; Department of Medicine, Columbia University Irving Medical Center, New York, NY 10032, USA; Department of Epidemiology, Columbia University Irving Medical Center, New York, NY 10032, USA; Division of Psychiatry, University College London, London W1T 7NF, UK; Department of Psychiatry and Behavioral Sciences, Stanford University, Stanford, CA 94305, USA; Davis School of Gerontology, University of Southern California, Los Angeles, CA 90089, USA; Institute of Cognitive Neuroscience, University College London, London WC1N 3AZ, UK; Division of Psychiatry, University College London, London W1T 7NF, UK

**Keywords:** neuropsychiatric, dementia, Alzheimer, autonomic, parasympathetic

## Abstract

Agitation in Alzheimer’s disease is common and may be related to impaired emotion regulation capacity. Heart rate variability, a proposed index of autonomic and emotion regulation neural network integrity, could be associated with agitation propensity in Alzheimer’s disease. We used the Atherosclerosis Risk in Communities Study cohort data, collected over seven visits spanning over two decades, to investigate whether heart rate variability (change) was associated with agitation risk in individuals clinically diagnosed with dementia due to Alzheimer’s disease. Agitation (absence/presence) at Visit 5, the primary outcome, was based on the Neuropsychiatric Inventory agitation/aggression subscale, or a composite score comprising the total number of agitation/aggression, irritability, disinhibition and aberrant motor behaviour subscales present. Visit 1–5 heart rate variability measures were the log-transformed root mean square of successive differences in R–R intervals and standard deviation of normal-to-normal R–R intervals obtained from resting, supine, standard 12-lead ECGs. To aid interpretability, heart rate variability data were scaled such that model outputs were expressed for each 0.05 log-unit change in heart rate variability (which approximated to the observed difference in heart rate variability with every 5 years of age). Among 456 participants who had dementia, 120 were clinically classified to have dementia solely attributable to Alzheimer’s disease. This group showed a positive relationship between heart rate variability and agitation risk in regression models, which was strongest for measures of (potentially vagally mediated) heart rate variability change over the preceding two decades. Here, a 0.05 log-unit of heart rate variability change was associated with an up to 10-fold increase in the odds of agitation and around a half-unit increase in the composite agitation score. Associations persisted after controlling for participants’ cognitive status, heart rate (change), sociodemographic factors, co-morbidities and medications with autonomic effects. Further confirmatory studies, incorporating measures of emotion regulation, are needed to support heart rate variability indices as potential agitation propensity markers in Alzheimer’s disease and to explore underlying mechanisms as targets for treatment development.

## Teaser

Liu *et al.* report that individuals clinically diagnosed with dementia due to Alzheimer’s disease showed a positive association between heart rate variability and risk of developing agitation. Specifically, compared to non-agitated individuals, individuals with agitation showed reduced longitudinal decline in heart rate variability over 22–26 years of follow-up.

## Graphical Abstract

**Graphical Abstract:**
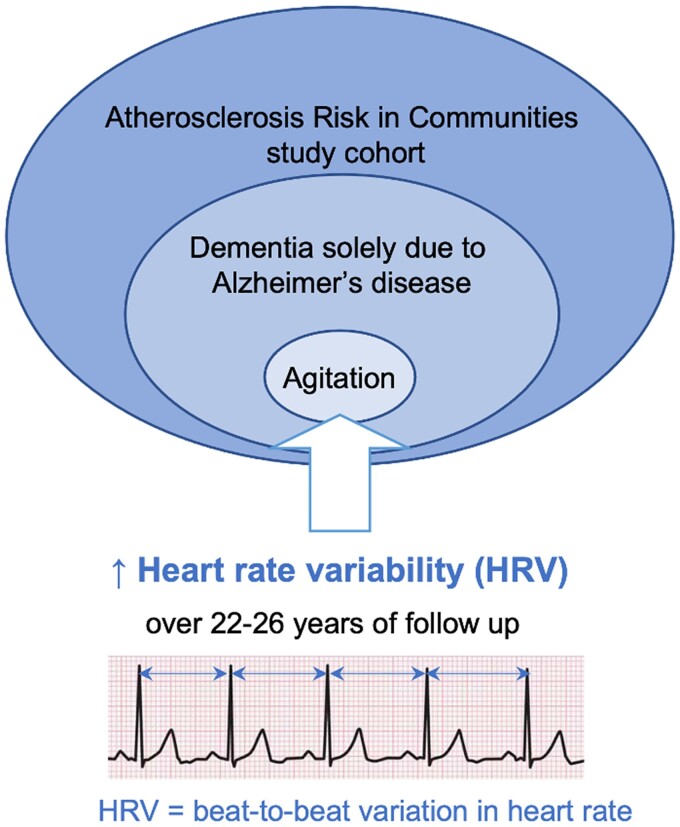

## Introduction

Agitation, defined as sustained, observed or inferred evidence of emotional distress associated with excessive motor activity, verbal aggression or physical aggression,^1^ is a common, distressing and difficult-to-treat neuropsychiatric syndrome in dementia. It affects around 30% of community-dwelling adults^2^ and 80% of nursing home residents with Alzheimer’s disease,^3^ the most common cause of dementia. Alzheimer’s disease–related disruption of neurotransmitter systems and neural networks underlying emotion regulation may contribute to the development of agitation.^4,5^ However, insufficient understanding of the neurobiology of agitation and the involvement of multiple, overlapping neurotransmitter systems have been barriers to the identification of safer and more effective prevention and treatment strategies.

Heart rate variability (HRV), the beat-to-beat variation in heart rate, is proposed to provide an objective measure of emotion regulation capacity. This is because it is believed to index the integrity of overlapping neural networks, comprising prefrontal cortical, limbic and brainstem regions, involved in both central autonomic nervous system and ‘top-down’ self-regulation processes.^6,7^ HRV is regulated by the involuntary (i.e. autonomic) nervous system with parasympathetic dominance,^8^ and the effective dynamic regulation of vagal tone, as generally indexed by higher HRV, has been proposed to underlie the adaptive capacity of the autonomic nervous system in response to external stimuli.^9^ In line with this, previous studies have shown that lower HRV is generally related to greater levels of psychopathology^10,11^ and worse cognitive function.^12,13^ Given that neurodegenerative disease processes can reduce the integrity of the central autonomic network^14,15^ and are associated with lower HRV,^13,16^ differences in HRV might reflect variability in executive function and emotion regulation capacity in individuals with dementia, which are processes hypothesized to underlie development of neuropsychiatric symptoms such as agitation.^5,17^ To understand its potential role in Alzheimer’s disease as a marker of agitation propensity and that of autonomic dysfunction as a target of treatment, it is important to assess whether HRV shows an association with the development of agitation.

With the availability of repeat measures of HRV over the mid- to late-life transition period, adjudicated diagnoses of dementia in late-life and assessment of agitation, the Atherosclerosis Risk in Communities (ARIC) Study cohort provides a unique opportunity to examine the relationship between longitudinal HRV and agitation in dementia. This study aims to analyse the relationship between cross-sectional HRV and HRV change over preceding visits with agitation point prevalence in Alzheimer’s disease. We tested our prediction that individuals with Alzheimer’s disease and agitation, hypothesized to have reduced self-regulatory capacity, would also have lower HRV and/or a larger decline in HRV over time compared to Alzheimer’s disease individuals without agitation.

## Materials and methods

### ARIC Study design

The ARIC Study (https://sites.cscc.unc.edu/aric/) is a prospective epidemiological cohort study conducted in four US communities (Forsyth County, NC; Jackson, MS; the northwest suburbs of Minneapolis, MN; and Washington County, MD). The ARIC Study enrolled 15 792 participants aged 45–64 years between 1987 and 1989,^18^ who have been followed up over seven visits up to 2020. Participants were examined at baseline (Visit 1), then between 1990–92 (Visit 2), 1993–95 (Visit 3), 1996–98 (Visit 4), 2011–13 (Visit 5), 2016–17 (Visit 6) and 2018–19 (Visit 7). Participants were also followed up by telephone call annually (1988–2011) or semi-annually (from 2012) for updates on selected health items.

The ARIC dementia adjudication procedures have been described previously.^19^ In summary, beginning at Visit 5, mild cognitive impairment (MCI) and dementia were both adjudicated by an expert panel of neurologists and neuropsychologists who reviewed the in-person cognitive assessment and other health data. Additional dementia cases were ascertained through data collected from annual follow-up telephone interviews, informant interviews and hospital discharge or death certificate codes. For participants who attended the in-person cohort exam, the Neuropsychiatric Inventory (NPI) was collected through informant interviews, and participant’s cognitive status (normal, MCI, dementia or undetermined), and aetiologic diagnosis if diagnosed with MCI or dementia, was determined. Reviewers could classify participants as having more than one dementia aetiology, and the agreement of two reviewers was required to designate a classification as primary. Thus, those diagnosed with MCI or dementia were given an aetiologic diagnosis of pure Alzheimer’s disease, Alzheimer’s disease with cerebrovascular disease (CVD), Alzheimer’s disease with Lewy body dementia (LBD), Alzheimer’s disease with other, pure CVD, CVD with Alzheimer’s disease, CVD with LBD, CVD with other, other or unknown. They were also given a primary aetiologic diagnosis of Alzheimer’s disease, CVD, LBD, depression, other major psychiatric disorder, alcohol related, medication related, other neurodegenerative disorder (e.g. progressive supranuclear palsy, corticobasal syndrome, Huntington’s disease and HIV dementia), trauma related, systemic disorder or cognitive disorder of uncertain aetiology. For the diagnosis of Alzheimer’s disease–related MCI or dementia, reviewers followed National Institute on Aging–Alzheimer’s Association criteria.^20,21^ Aetiologic diagnoses were only available at Visit 5, and MCI diagnosis, aetiologic diagnoses and NPI scores were assigned only for individuals who were seen in person. The ARIC Study protocol was approved by the institutional review board of each participating centre, and informed consent was obtained from participants at each visit.

### Participant selection criteria

**Figure 1:**
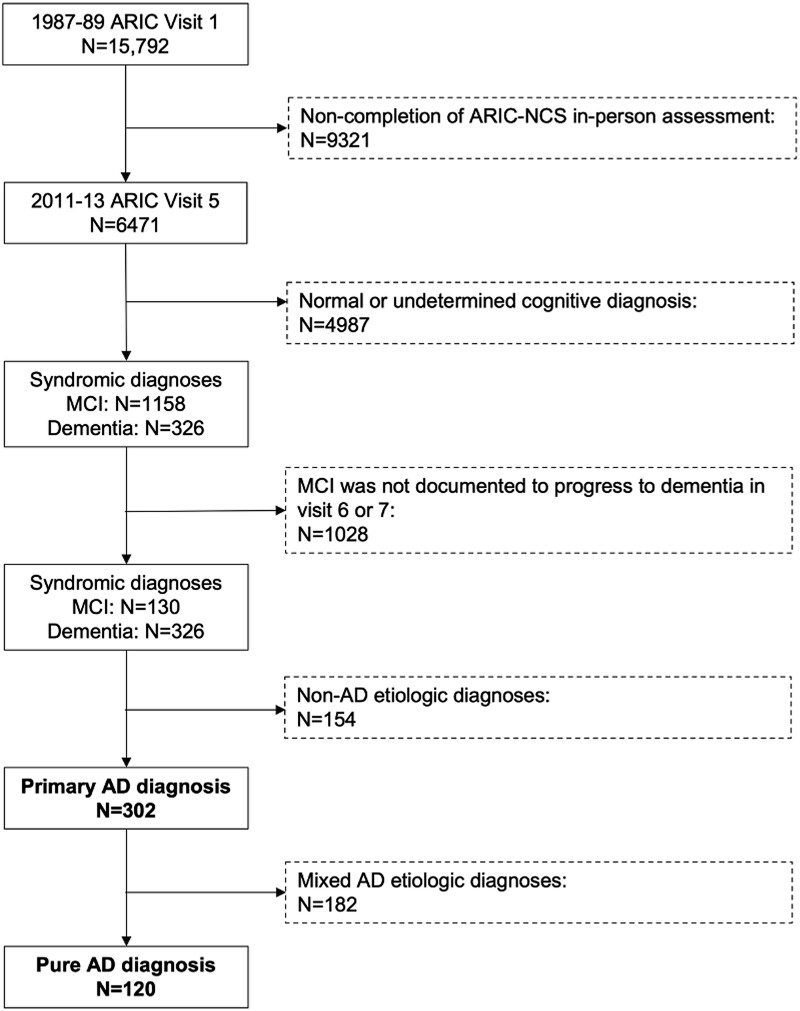
**Flowchart of study exclusions and analytic sample.** Exclusion criteria are shown in the dashed boxes on the right. The analytic samples (‘primary Alzheimer’s disease’ and ‘pure Alzheimer’s disease’) are highlighted in bold.

Considering prior data that suggest that around a third of MCI patients do not progress to dementia^22,23^ and may not have an underlying neurodegenerative disorder, we restricted analyses to participants diagnosed with dementia or with MCI that subsequently progressed to dementia, i.e. individuals diagnosed with MCI at Visit 5 and who did not have a diagnosis of dementia at Visit 6 or 7 were excluded. Thus, all participants with a primary aetiological diagnosis of Alzheimer’s disease at Visit 5, who had dementia or MCI that subsequently progressed to dementia (‘primary Alzheimer’s disease’), which included a subgroup with a clinical aetiological diagnosis of ‘pure Alzheimer’s disease’ (i.e. dementia was solely attributed to Alzheimer’s disease and not a mixed dementia), were included in the study (*n* = 302; Fig. 1). This included individuals who did not receive in-person assessments at Visit 6 or 7.

### HRV measures

Two time domain HRV measures were obtained at Visits 1–5 from resting, supine, 10-s, standard 12-lead ECGs: root mean square of successive differences in R–R intervals (RMSSD) and standard deviation of normal-to-normal R–R intervals (SDNN).^24^ Whilst RMSSD is the primary time domain measure used to estimate the vagally mediated changes reflected in HRV, both parasympathetic and sympathetic nervous system activities contribute to SDNN.^25^ All 12 leads were used for HRV analysis, and ECGs with the following abnormalities were flagged and excluded before computing HRV metrics: poor quality grade (based on noise/artefact/interference; overall drift; beat-to-beat drift); fewer than 5 or <50% normal–normal R–R intervals; electronic pacing; Wolff–Parkinson–White syndrome; atrial fibrillation or flutter; secondary atrioventricular block; tertiary atrioventricular block; Mobitz type II; Wenckebach phenomenon; premature beats; wandering atrial pacemaker; ventricular tachycardia; supraventricular rhythm; supraventricular tachycardia; and pause. Only time domain (SDNN and RMSSD) measures from 10-s ECG recordings were obtained at Visits 1–5 and were thus available for longitudinal analysis; we did not include time and frequency domain measures from longer 2- or 6-min recordings as these were only available at Visit 1 or 4.

The measures were log transformed to achieve a normal distribution.^25^ Normal distributions of mean logSDNN and logRMSSD values across Visits 1–5 were confirmed using Shapiro–Wilk tests. Given the relatively narrow range and scale of the resulting logHRV (logRMSSD or logSDNN distributions; Supplementary Tables 1 and 2), we optimized the interpretability of our findings by scaling the log-transformed values such that model estimates were expressed as 0.05 log-unit increases in HRV. A 0.05 log-unit change in HRV approximates the observed difference in HRV with each 5 years of age in the study population (e.g. −0.046 for logRMSSD and −0.052 for logSDNN, equivalent to −1.04 for RMSSD and −0.99 for SDNN in the ‘pure Alzheimer’s disease’ group).

### Agitation measures

The NPI was administered at Visits 5, 6 and 7. An individual with dementia or MCI was defined as having agitation if the agitation/aggression subscale item was marked as present. As agitation has been defined as emotional distress associated with excessive motor overactivity, or verbal or physical aggression,^1^ we also investigated a broader, composite definition of agitation, which was the total number of subscale items out of agitation/aggression, irritability, disinhibition and aberrant motor behaviour, marked as present.^26,27^ Descriptions of these NPI subscales are shown in Table 1.

**Table 1:** Description of the NPI agitation/aggression subscale and three additional subscales that contributed to a broader agitation measure

NPI subscale	Description
Agitation/aggression	Is the patient resistive to help from others at times, or hard to handle?
Disinhibition	Does the patient seem to act impulsively, for example, talking to strangers as if he/she knows them, or saying things that may hurt people’s feelings?
Irritability/lability	Is the patient impatient and cranky? Does he/she have difficulty coping with delays or waiting for planned activities?
Motor disturbance	Does the patient engage in repetitive activities such as pacing around the house, handling buttons, wrapping string, or doing other things repeatedly?

To explore whether any observed association between HRV and agitation was related to a mood disorder, psychosis or a dysexecutive syndrome, we also investigated three other composite NPI subscale scores proposed to represent major neuropsychiatric symptom clusters in Alzheimer’s disease.^26^ These NPI factors were ‘mood’ (total number of NPI-rated depression, anxiety and irritability subscale items present), ‘psychosis’ (total number of NPI-rated hallucination and delusion items present) and ‘frontal’ (total number of NPI-rated elation, apathy, disinhibition and irritability items present).

### Statistical analyses

Analyses were conducted in participants with ‘primary Alzheimer’s disease’ (*n* = 302) and repeated for the ‘pure Alzheimer’s disease’ subgroup (*n* = 120). We ran separate models of agitation (NPI subscale or composite total) on logHRV.

Due to a high proportion of missing NPI data observed at Visits 6 and 7 (up to 95% in the ‘primary Alzheimer’s disease’ group and 92% in the ‘pure Alzheimer’s disease’ subgroup, Supplementary Tables 1 and 2, respectively), which was likely related to fewer cases of dementia being defined in-person during these visits (NPI scores were assigned only for individuals who were seen in person), we assessed agitation point prevalence at Visit 5 only. The associations between agitation and Visit 5 logHRV or Visit 1–5 change in logHRV were assessed using simple (logistic for the binary NPI subscale agitation outcome and linear for the continuous composite agitation outcome) regression models. We confirmed an assumed linear association between logHRV and agitation by visually inspecting a scatterplot between logHRV and the log odds of agitation.

Visit 1–5 change in logHRV (or heart rate) was defined as individual slope coefficients, comprising random and fixed effects, from a mixed-effects linear regression model where logHRV (or heart rate) was the dependent variable and follow-up years since Visit 1 (baseline) was the independent variable. Mixed-effects models can accommodate the correlation between repeated measures due to unobserved inter-individual heterogeneity by incorporating random effects. They can also account for unequal follow-up intervals by including time as a continuous variable. Models with random intercepts and slopes and correlated random effects provided optimal model fit.

Three sets of regressions were conducted for each model: (i) unadjusted; (ii) adjusted for heart rate (or heart rate change when assessing logHRV change); and (iii) adjusted for heart rate (or heart rate change) and separate sociodemographic factors. These factors were Visit 5 age in years, Mini-Mental State Examination (MMSE) score, sex, a combined race–centre variable (due to the disparate distribution of race groups across the ARIC centres, categorized as Forsyth County-White, Jackson-Black, Minneapolis-White, and Washington County-White) and presence/absence of comorbidities (separate variables for hypertension and diabetes). We adjusted models for heart rate as this has been reported to influence the cardiovascular predictive value of HRV and its reproducibility,^28^ and higher resting heart rate may be a risk factor for dementia and faster cognitive decline.^29^ Log odds were converted to odds ratios to aid interpretability. All analyses were performed in R version 4.0.2, and the relationships between variables were tested at a significance level of *α* = 0.05 (two-tailed).

### Missing data

The number of missing observations was reported. Complete case analysis (i.e. listwise deletion) was performed for the simple logistic and linear regression models, whereas the mixed-effects models incorporated all available logHRV data using maximum likelihood estimation.

To assess the range of uncertainty due to missing NPI-rated agitation subscale or logHRV data at Visit 5, we employed a ‘best-worse-case’ sensitivity analysis by replacing missing dichotomous agitation subscale data with either 0 (absent) or 1 (present), and for the Visit 5 logHRV analysis, we also explored replacing missing continuous logHRV data with mean logHRV values ±2 SD.^30^

### *Post hoc* analyses

As certain medications may influence relationships between agitation and HRV (e.g. antipsychotics used to treat agitation can lower HRV),^31,32^ we examined whether antipsychotic, serotonin noradrenergic reuptake inhibitor (SNRI), mirtazapine, acetylcholinesterase inhibitor (AChEI) and oral or parenteral β-blocker use influenced findings in the ‘pure Alzheimer’s disease’ subgroup. Since no ‘pure Alzheimer’s disease’ participants used SNRIs or mirtazapine at any visit (Supplementary Table 2), only binary indicators of antipsychotic, AChEI and β-blocker use were added to the final adjusted models. The (0;1) indicators at Visit 5 were included in the Visit 5 logHRV models, and their totals across Visits 1–5 (ranging from 0, i.e. no usage at any visit, to 5, i.e. usage at all visits) were included in the logHRV change models.

We examined whether observed logHRV differences between agitated and non-agitated Alzheimer’s disease individuals were related to a putative measure of peripheral sympathetic hyperactivity, comprising a composite (average) of *Z*-scores for systolic blood pressure, creatinine, fasting glucose and C-reactive protein levels, which were available for >75% of the study sample at Visits 2 and 5.

To assess possible attrition bias related to the exposure, we explored whether there was an association between baseline (Visit 1) logHRV and Visit 5 in-person cognitive assessment non-attendance, or non-attendance due to death, using logistic regression models for all individuals with baseline HRV measures.

Lastly, to further explore and contextualize observed logHRV differences between agitated and non-agitated individuals with ‘pure Alzheimer’s disease’, we compared logHRV change over Visits 1–5 between these subgroups, as well as between either subgroup and a comparison group comprising individuals diagnosed as cognitively normal at Visits 5, 6 and 7 (i.e. they did not develop MCI or dementia during the study period) and had at least one HRV measurement (*n* = 1270). Comparisons were adjusted for heart rate or heart rate change and sociodemographic factors as previously described (Visit 5 age, sex, MMSE, race-centre, hypertension and diabetes) using analysis of covariance. Any significant differences between groups were assessed using *post hoc* Tukey tests.

## Results

### Characteristics of subjects included in analyses

At Visit 5, 456 individuals received an adjudicated clinical diagnosis of dementia or MCI that subsequently progressed to dementia (Fig. 1). Most (*n* = 302, 66%) received a primary aetiological diagnosis of Alzheimer’s disease (‘primary Alzheimer’s disease’), of whom 40% (*n* = 120) were clinically considered to have dementia solely attributable to Alzheimer’s disease; in other words, they did not have a mixed dementia (‘pure Alzheimer’s disease’). Other primary diagnoses adjudicated at Visit 5 included CVD (*n* = 100, 22%), LBD (*n* = 23, 5%), depression (*n* = 3, <1%), other major psychiatric disorders (*n* = 1, <1%) and uncertain aetiology (*n* = 27, 6%). Of the 456 individuals who had or progressed to dementia, Alzheimer’s disease was explicitly recorded not to be a primary or secondary aetiologic diagnosis in *n* = 22 [5%, composed of pure CVD (*n* = 11) and CVD with LBD (*n* = 11)].

Detailed characteristics across Visits 1–5 for the two study populations who developed dementia during the study, i.e. those who had ‘primary Alzheimer’s disease’ (*n* = 302) and a subgroup with ‘pure Alzheimer’s disease’ (*n* = 120), are shown in Supplementary Tables 1 and 2, respectively. The ‘pure Alzheimer’s disease’ subgroup characteristics by agitation status are shown in Table 2 for Visit 5 and Supplementary Table 3 for baseline (Visit 1) characteristics. Participants were followed up for up to 26.4 years from baseline. Characteristics of a cognitively unimpaired comparison group, used in *post hoc* analyses (*n* = 1270), are shown in Supplementary Table 4.

**Table 2:** Visit 5 characteristics of the ‘pure Alzheimer’s disease’ subgroup by NPI agitation status

Visit 5 characteristics	NPI agitation present (*n* = 26)	NPI agitation absent (*n* = 85)
Age in years [mean (SD)]	78.9 (5.2)	78.2 (5.1)
Diagnostic group (%)		
MCI	4 (15)	39 (46)
Dementia	22 (85)	46 (54)
MMSE [mean (SD)]	20.8 (5.9)	21.2 (6.4)
Agitation composite total score		
0	0	64 (75)
1	10 (38)	14 (16)
2	9 (35)	6 (7)
3	6 (23)	1 (1)
4	1 (4)	0
HRV [mean (SD), range]		
RMSSD^a^	20.1 (12.6–33.8), 2.5–83.8 (*M* = 7)	14.0 (8.2–25.2), 2.1–103.5 (*M* = 38)
SDNN^a^	16.2 (11.8–30.6), 2.2–63.1 (*M* = 7)	12.9 (8.4–20.1), 1.2–51.7 (*M* = 38)
logRMSSD	3.0 (0.8), 0.9–4.4 (*M* = 7)	2.6 (0.7), 0.8–4.6 (*M* = 38)
logSDNN	2.9 (0.8), 0.8–4.1 (*M* = 7)	2.5 (0.7), 0.2–3.9 (*M* = 38)
logRMSSD change^b^	0.0002 (0.02), −0.04 to 0.05	−0.01 (0.02), −0.1 to 0.04 (*M* = 1)
logSDNN change^b^	−0.004 (0.02), −0.04 to 0.04	−0.01 (0.02), −0.1 to 0.03 (*M* = 1)
Heart rate (b.p.m.)		
Mean (SD)	62.1 (9.7) (*M* = 4)	60.8 (12.1) (*M* = 30)
Heart rate change^b^ [mean (SD)]	−0.09 (0.26)	−0.18 (0.25)
Comorbidities (%)		
Diabetes	10 (40) (*M* = 1)	26 (38) (*M* = 16)
Hypertension	16 (67) (*M* = 2)	54 (69) (*M* = 7)
Prescribed medication(s) (%)		
Antipsychotics	0	3 (4)
SNRIs	0	0
AChEIs	7 (27)	21 (25)
β-Blockers	5 (19)	23 (27)

NPI agitation subscale data, obtained at Visit 5, were missing for nine participants. Any missing values were reported (*M* = number of missing data points).

^a^For non-normally distributed RMSSD and SDNN data, median (interquartile range) values are shown.

^b^Change in logHRV or heart rate was calculated as the slope coefficient using mixed-effects linear regression models where follow-up time in years since baseline (Visit 1) was the independent variable.

### Agitation and HRV at Visit 5

We observed a positive relationship between Visit 5 HRV and agitation risk in the ‘pure Alzheimer’s disease’ subgroup but not in the larger ‘primary Alzheimer’s disease’ group. This was specific to the association between the logRMSSD (but not logSDNN) HRV measure and agitation after adjusting for heart rate and sociodemographic factors. A 0.05 unit increase in logRMSSD (equivalent to the mean HRV difference over 5 years in the ‘pure Alzheimer’s disease’ group) was associated with 6% higher odds of NPI subscale agitation {odds ratio [95% confidence interval (CI)] = 1.06 [1.01–1.13]; Table 3} and a 0.02 (95% CI 0.002–0.04) unit increase in the composite agitation score (Supplementary Table 5).

**Table 3:** Associations between Visit 5 logHRV (logRMSSD or logSDNN) and NPI subscale agitation for the ‘pure Alzheimer’s disease’ group

Logistic regression models	Log odds (95% CI)	Odds ratio (95% CI)
logRMSSD		
Unadjusted	0.04 (−0.001 to 0.08)	1.04 (0.998–1.08)
Adjusted 1	**0.06 (0.01–0.12)**	**1.06 (1.01–1.11)**
Adjusted 2	**0.06 (0.007–0.12)**	**1.06 (1.01–1.13)**
logSDNN		
Unadjusted	0.04 (−0.002 to 0.08)	1.04 (0.998–1.08)
Adjusted 1	**0.04 (0.005–0.09)**	**1.05 (1.00–1.10)**
Adjusted 2	0.04 (−0.003 to 0.09)	1.04 (0.996–1.10)

Statistically significant (*P* < 0.05) results are highlighted in bold. Odds are expressed for each 0.05 logHRV unit change, approximately corresponding to the observed HRV difference by 5 years of age. The relationship between logHRV and agitation was adjusted for heart rate (adjusted 1 models) and heart rate and sociodemographic factors (Visit 5 age, sex, MMSE, race–centre, hypertension and diabetes; adjusted 2 models).

### Agitation and HRV change over Visits 1–5

We also observed a positive relationship between HRV change over Visits 1–5 and agitation risk in the ‘pure Alzheimer’s disease’ subgroup but not the larger ‘primary Alzheimer’s disease’ group. In fully adjusted models, a 0.05 unit increase in logHRV change (equivalent to the observed mean HRV difference over 5 years within the ‘pure Alzheimer’s disease’ group) was associated with up to a 10-fold increase in the odds of NPI subscale agitation [odds ratio (95% CI) for logRMSSD = 10.44 (2.23–70.42); for logSDNN = 6.55 (1.48–41.08); Table 4] and around a half unit increase in the composite agitation score [regression coefficient (95% CI) for logRMSSD = 0.61 (0.17–1.05); for logSDNN = 0.51 (0.04–0.97); Table 5]. There was a positive correlation between HRV and the ‘frontal’ factor score, with only the logRMSSD measure surviving adjustment for heart rate and sociodemographic factors [regression coefficient (95% CI) = 0.50 (0.04–0.97); Table 5].

**Table 4:** Associations between logHRV change (logRMSSD or logSDNN) over Visits 1–5 and NPI subscale agitation for the ‘pure Alzheimer’s disease’ group

Logistic regression models	Log odds (95% CI)	Odds ratio (95% CI)
logRMSSD change		
Unadjusted	**1.40 (0.30–2.64)**	**4.07 (1.35–14.04)**
Adjusted 1	**2.19 (0.86–3.75)**	**8.98 (2.37–42.51)**
Adjusted 2	**2.35 (0.80–4.25)**	**10.44 (2.23–70.42)**
logSDNN change		
Unadjusted	**1.57 (0.29–2.99)**	**4.78 (1.34–19.96)**
Adjusted 1	**1.96 (0.57–3.55)**	**7.07 (1.76–34.83)**
Adjusted 2	**1.88 (0.39–3.72)**	**6.55 (1.48–41.08)**

Statistically significant (*P* < 0.05) results are highlighted in bold. Odds are expressed for each 0.05 logHRV unit change, approximately corresponding to the observed HRV difference by 5 years of age. The relationship between logHRV change and agitation was adjusted for heart rate change (adjusted 1 models) and heart rate change and sociodemographic factors (Visit 5 age, sex, MMSE, race–centre, hypertension and diabetes; adjusted 2 models).

**Table 5:** Associations between logHRV change (logRMSSD or logSDNN) over Visits 1–5 and agitation or frontal composite scores for the ‘pure Alzheimer’s disease’ group

Linear regression models	Unstandardized (*B*) regression coefficients (95% CI)
Agitation composite total score	
logRMSSD change	
Unadjusted	**0.47 (0.06–0.89)**
Adjusted 1	**0.63 (0.19–1.06)**
Adjusted 2	**0.61 (0.17–1.05)**
logSDNN change	
Unadjusted	**0.52 (0.06–0.99)**
Adjusted 1	**0.59 (0.12–1.06)**
Adjusted 2	**0.51 (0.04–0.97)**
Frontal composite total score	
logRMSSD change	
Unadjusted	**0.51 (0.12–0.90)**
Adjusted 1	**0.58 (0.17–1.00)**
Adjusted 2	**0.50 (0.04–0.97)**
logSDNN change	
Unadjusted	**0.46 (0.02–0.91)**
Adjusted 1	**0.48 (0.03–0.93)**
Adjusted 2	0.36 (−0.13 to 0.86)

Statistically significant (*P* < 0.05) results are highlighted in bold. Regression coefficients are expressed for each 0.05 logHRV unit change, approximately corresponding to the observed HRV difference by 5 years of age. The relationship between logHRV change and agitation was adjusted for heart rate change (adjusted 1 models) and heart rate change and sociodemographic factors (Visit 5 age, sex, MMSE, race–centre, hypertension and diabetes; adjusted 2 models). Change in HRV or heart rate was calculated as the slope coefficient using mixed-effects models where follow-up time (years) since baseline (Visit 1) was the independent variable.

### Missing data

A small proportion of individuals with a ‘primary Alzheimer’s disease’ (*n* = 19, 6.3%) or ‘pure Alzheimer’s disease’ (*n* = 9, 7.5%) had missing NPI data at Visit 5. A larger proportion of individuals with ‘primary Alzheimer’s disease’ (*n* = 134, 44.3%) or ‘pure Alzheimer’s disease’ (*n* = 51, 42.5%) had missing HRV data at Visit 5 (Supplementary Tables 1 and 2). This resulted in the listwise deletion of 54 missing observations in the Visit 5 HRV models, and only 10 missing observations were deleted in the HRV change models.

The replacement of missing NPI subscale agitation data with either 0 (absent) or 1 (present) did not qualitatively change the previously observed relationships with Visit 5 HRV and HRV change in ‘pure Alzheimer’s disease’ individuals (Table 6). However, the cross-sectional relationship between Visit 5 RMSSD and NPI subscale agitation was no longer statistically significant after missing Visit 5 HRV data were replaced with mean logHRV + 2 SD or −2 SD.

**Table 6:** ‘Best-worst-case’ associations between logHRV and NPI subscale agitation in the ‘pure Alzheimer’s disease’ group

Logistic regression models	Log odds (95% CI)	Odds ratio (95% CI)
Agitation present		
Visit 5 logRMSSD	**0.06 (0.009–0.12)**	**1.06 (1.01–1.12)**
Visit 5 logSDNN	0.04 (−0.002 to 0.09)	1.04 (0.998–1.09)
logRMSSD change^a^	**1.71 (0.41–3.28)**	**5.52 (1.50–26.69)**
logSDNN change^a^	**1.58 (0.23–3.23)**	**4.88 (1.26–25.39)**
Agitation absent		
Visit 5 logRMSSD	**0.05 (0.004–0.11)**	**1.05 (1.00–1.12)**
Visit 5 logSDNN	0.04 (−0.006 to 0.08)	1.04 (0.99–1.09)
logRMSSD change^a^	**2.51 (0.94–4.42)**	**12.26 (2.56–83.00)**
logSDNN change^a^	**1.97 (0.44–3.83)**	**7.16 (1.55–45.91)**
logHRV mean + 2 SD		
Visit 5 logRMSSD	0.02 (−0.01 to 0.06)	1.02 (0.99–1.06)
Visit 5 logSDNN	0.02 (−0.01 to 0.05)	1.02 (0.99–1.06)
logHRV mean − 2 SD		
Visit 5 logRMSSD	0.04 (0.001–0.08)	1.04 (1.00–1.09)
Visit 5 logSDNN	0.03 (−0.007 to 0.06)	1.03 (0.99–1.07)

Statistically significant (*P* < 0.05) results are highlighted in bold. Odds are expressed for each 0.05 logHRV unit change, approximately corresponding to the observed HRV difference by 5 years of age. Missing dichotomous NPI subscale agitation data were replaced with either 1 (agitation present) or 0 (agitation absent). Missing Visit 5 logHRV data were replaced with mean logHRV ± 2 SD. Model estimates were adjusted for heart rate (change) and sociodemographic factors (Visit 5 age, sex, MMSE, race–centre, hypertension and diabetes; equivalent to adjusted 2 models).

^a^Change in HRV or heart rate was calculated as the slope coefficient using mixed-effects models where follow-up time (years) since baseline (Visit 1) was the independent variable.

### *Post hoc* analyses

The inclusion of antipsychotic, AChEI and β-blocker use in the adjusted models did not change the overall findings. There was no relationship between a putative measure of peripheral sympathetic activity (either at Visit 5 or the difference between Visits 2 and 5) and logHRV change slope coefficients or agitation status. We did not find a significant relationship between Visit 1 (baseline) HRV and Visit 5 non-attendance at the in-person cognitive assessment or dropout due to death.

**Figure 2:**
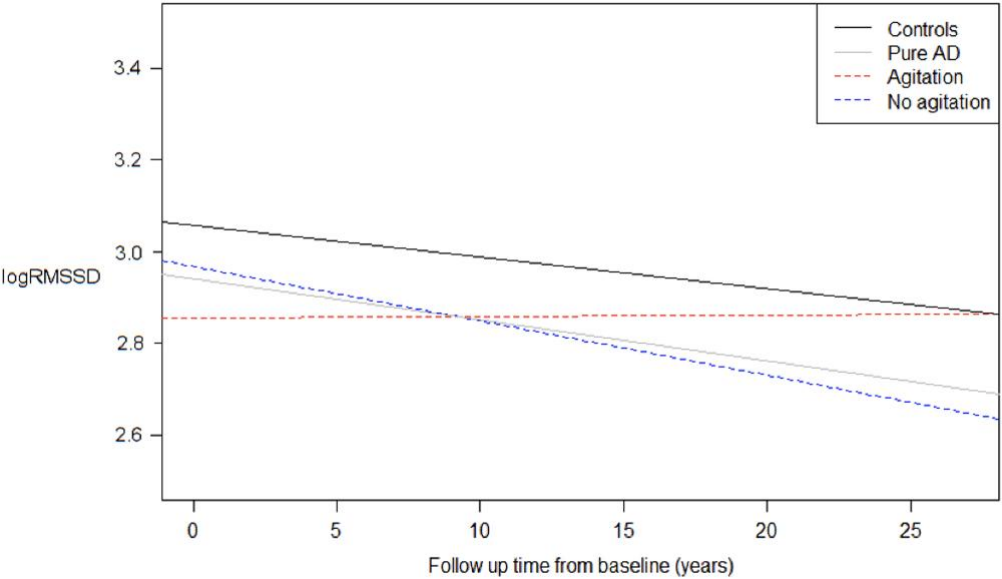
**logRMSSD slopes from unadjusted mixed-effects models for ‘pure Alzheimer’s disease’ (*n* = 120) and a comparison group (*n* = 1270) who were cognitively unimpaired.** Slopes were plotted using regression coefficient and intercept values from the unadjusted mixed-effects linear regression models, where logHRV was the dependent variable and time in years from baseline (Visit 1) was the independent variable. Original (unscaled) logRMSSD values are displayed. The slope (logRMSSD change) and intercept (Visit 1) values for the pure Alzheimer’s disease and comparison groups, from unadjusted mixed-effects models, are shown in Supplementary Tables 2 and 4, respectively. The unadjusted slope (and intercept) value for the pure Alzheimer’s disease with agitation group (*n* = 26) was 0.00024 (2.85) and for the non-agitated group (*n* = 85) was −0.012 (2.96).

‘Pure Alzheimer’s disease’ individuals with agitation had higher logHRV slope coefficients compared to those without agitation (adjusted mean difference for logRMSSD = 0.018, *P* = 0.002; for logSDNN = 0.014, *P* = 0.01) but not compared to cognitively unimpaired individuals. In contrast, non-agitated ‘pure Alzheimer’s disease’ individuals had lower logHRV slope coefficients compared to the cognitively unimpaired group (adjusted mean difference for logRMSSD = 0.009, *P* = 0.001; for logSDNN = 0.008, *P* = 0.002). There were no adjusted mean group differences in Visit 5 heart rate or heart rate change over Visits 1–5 between the comparison group and ‘pure Alzheimer’s disease’. Unadjusted and non-scaled logRMSSD slopes and intercepts from the mixed models are plotted in Fig. 2.

## Discussion

We observed a positive relationship between HRV, particularly HRV change over the preceding two decades, and agitation risk in individuals clinically diagnosed with dementia solely attributed to Alzheimer’s disease. A general decline in HRV is observed with increasing age. However, compared to non-agitated Alzheimer’s disease individuals, agitated individuals showed reduced overall longitudinal decline in HRV measured over 22–26 years of follow-up. Our findings are the first identification of a potential autonomic marker for, and implicating the role of specific neurobiological processes in, agitation propensity in Alzheimer’s disease.

Although it was the opposite direction to what we had predicted, a possible explanation for the positive relationship observed in ‘pure Alzheimer’s disease’ (and not Alzheimer’s disease as part of a mixed dementia) is that this group showed a stronger influence of Alzheimer’s disease–related locus coeruleus (LC)–noradrenergic system degeneration on autonomic system dysfunction. The LC, the brain’s main source of noradrenaline (NA), is one of the earliest brain regions to be affected by Alzheimer’s disease–related pathology.^33^ It upregulates cortical arousal, increases sympathetic activity and reduces parasympathetic activity in response to stress.^34^ Consistent with the prediction that Alzheimer’s disease–related loss of LC neurons results in increased parasympathetic and reduced sympathetic activity,^35^ studies have shown that reduced LC signal intensity on ‘neuromelanin-sensitive’ magnetic resonance images, a marker of Alzheimer’s disease–related neurodegeneration,^36^ is associated with higher HRV.^37,38^ This implies possible dissociation between higher HRV and better self-regulatory processes in individuals with more severe Alzheimer’s disease–related LC cell loss—a phenomenon that has also been observed in other psychiatric and autonomic disorders.^11,39^

Alternatively, compensatory neural activity may result in apparent ‘preserved’ autonomic function and less Alzheimer’s disease–related HRV decline than expected. For example, relative (compensatory) LC–NA system hyperactivity, secondary to Alzheimer’s disease–related LC cell loss, may predispose to agitation via impaired cortical and subcortical regulation of behaviour.^40^ Or there may be prefrontal cortical compensatory changes in response to peripheral sympathetic hyperactivity, although we were unable to find differences in putative markers of sympathetic activity. Potential associated mechanisms could include impaired interpretation of autonomic signals or interoceptive prediction errors that relate to emotion dysregulation.

Our findings are also consistent with an earlier study^41^ that found higher resting high-frequency HRV (which is highly correlated with RMSSD)^24,25^ in individuals with lower Alzheimer’s disease–related regional cortical thickness (i.e. greater disease severity), which was mediated by increased (possibly compensatory) functional MRI activation in anterior cingulate cortex, a region involved in autonomic and emotion regulation and interoception.^42,43^ The observed differences in logHRV slopes between agitated and non-agitated individuals in our study may at least partly explain the inconsistent findings of HRV in Alzheimer’s disease from previous studies.^16^

We observed a larger effect for HRV change over Visits 1–5 compared to Visit 5 HRV, suggesting that HRV ‘change’ over time may be a better indicator of agitation propensity as Alzheimer’s disease progresses, compared to a single measure of HRV. The relationship was more robust for RMSSD, considered an indicator of parasympathetic function, versus SDNN, a measure of mixed parasympathetic and sympathetic activity.^25^ This is important and consistent with the concept that the adaptive capacity of the autonomic system relies on the dynamic regulation of vagal tone.^9^ The observed relationship between increased HRV change and the frontal factor, but not mood or psychosis factors, in ‘pure Alzheimer’s disease’ individuals also supports the involvement of impaired frontal self-regulation in agitation propensity.^5^

### Missing data and limitations

As extreme logHRV values at Visit 5 resulted in some qualitatively different outcomes for the ‘pure Alzheimer’s disease’ subgroup, it is possible that the listwise deletion of missing HRV values biased outcomes for the Visit 5 cross-sectional analyses. Multiple random imputation was not conducted for these models as the amount of missing agitation subscale data was negligible (6%), and missing Visit 5 HRV data were substantial (44%).^30^ For the HRV change models, almost all HRV data were incorporated into the longitudinal mixed models (only one observation was deleted due to missingness across Visits 1–5).

Aetiologic diagnoses were clinically assigned without the availability of specific Alzheimer’s disease–related pathologic biomarkers; thus, possibly a number of participants were misdiagnosed and did not have ‘pure Alzheimer’s disease’. We did not compare our findings to a non-Alzheimer’s disease aetiology MCI/dementia subgroup as there was unlikely to be sufficient power (only 22 individuals were recorded not to have Alzheimer’s disease as a primary or secondary diagnosis). Although we attempted to account for medication use and physical comorbidities in the ‘pure Alzheimer’s disease’ subgroup, there was potential for residual confounding related to this. Potential survivorship bias is unlikely to be limited to ‘pure Alzheimer’s disease’, and baseline HRV was unrelated to Visit 5 in-person cognitive assessment non-attendance or dropout due to death, but we did not directly address participant attrition (due to death or dropout), so our findings may still have been subject to selection bias. A sensitivity analysis of only ‘pure Alzheimer’s disease’ participants who had dementia at Visit 5 (to account for potential selection bias related to attendance at Visit 6 or 7 in MCI participants) would likely lack sufficient power (*n* = 73).

Although earlier studies have reported on the potential validity of single ‘ultra-short’ 10-s HRV recordings, especially for RMSSD,^25,44,45^ and such recordings show expected relationships with age and cognition in our study,^46,47^ these may have been subject to higher measurement error compared to multiple or longer recordings.^24,48^ Although a 5-min ECG recording is most commonly employed to measure short-term HRV, ultra-short ECG recordings are increasingly employed in HRV studies as they represent an easy-to-obtain physiological measure that can be passively collected from individuals in research and/or clinical settings.^25,45^ The availability of ultra-short ECG recordings from large longitudinal cohorts such as ARIC represents an opportunity to test novel hypotheses, which may justify further validation and replication in other data sets and prospective studies. For example, only resting time domain HRV metrics from 10-s recordings were available at Visits 1–5 for longitudinal analysis, and the ‘raw’ data files were not available for re-analysis. Thus, future validation and replication studies are now needed to examine whether agitation in Alzheimer’s disease is associated with longer (e.g. 5 min), frequency domain or non-linear (e.g. SD1/SD2) HRV indices. Additionally, HRV reactivity in response to increased cognitive demand, proposed to be an indicator of impaired adaptive capacity and regulation of vagal tone, may provide additional insight into Alzheimer’s disease–related changes in autonomic function.^9,41^ Older individuals are more likely to experience abnormal cardiac conduction/rhythm abnormalities, which may elevate HRV,^49^ and although ECGs with abnormalities were excluded from analysis, it is possible this may have disproportionately affected the agitated Alzheimer’s disease group. Thus, it is possible that rather than true differences in autonomic function, erratic rhythms and cardiovascular disease might have contributed to the observed relationship between HRV and agitation risk in Alzheimer’s disease. Further research is needed to explore the precise mechanisms underlying the observed association. It is uncertain whether the same recording time applied to all ECG data, so HRV circadian rhythms may have affected our estimates.

In summary, we found evidence for a relationship between higher (potentially vagally mediated) HRV and agitation point prevalence in participants with Alzheimer’s disease dementia. It would be important to replicate this relationship in other data sets, alongside other measures of autonomic and emotion regulation and LC–NA system integrity. Further research is needed on the potential for HRV indices to be a marker of agitation propensity in Alzheimer’s disease and on underlying mechanisms as potential treatment targets in agitation.

## Supplementary Material

fcad269_Supplementary_Data

## Data Availability

Availability of data and detailed policies for accessing the Atherosclerosis Risk in Communities (ARIC) Study data can be found online (https://sites.cscc.unc.edu/aric/).
