# Supplementary material for: Heart rate variability and risk of agitation in Alzheimer’s disease: the Atherosclerosis Risk in Communities Study
Source: Brain Commun. 2023 Oct 13;5(6):fcad269. doi: 10.1093/braincomms/fcad269 (PMC10631859; doi:10.1093/braincomms/fcad269)
Supplement: fcad269_Supplementary_Data [file fcad269_supplementary_data.docx]

**Supplementary Files**

[Supplementary Table 1: Characteristics of the study population who had a primary etiological diagnosis of AD (*n* = 302). 1](#_Toc145498382)

[Supplementary Table 2: Characteristics of participants who were clinically diagnosed with dementia attributed solely to AD (‘pure AD’) (*n* = 120). 2](#_Toc145498383)

[Supplementary Table 3: Visit 1 (baseline) characteristics of the ‘pure AD’ subgroup by NPI agitation status 3](#_Toc145498384)

[Supplementary Table 4: Characteristics of a comparison group who were cognitively normal at visits 5, 6, and 7 (*n* = 1270). 4](#_Toc145498385)

[Supplementary Table 5: Associations between visit 5 logHRV (logRMSSD or logSDNN) and agitation composite scores in the ‘pure AD’ group. 5](#_Toc145498386)

### **Supplementary Table 1: Characteristics of the study population who had a primary etiological diagnosis of AD (*n* = 302).**

| **Visit** | **1** | **2** | **3** | **4** | **5** | **6** | **7** |
| --- | --- | --- | --- | --- | --- | --- | --- |
| *Diagnostic group* (%) |  |  |  |  |  |  |  |
| Normal | - | - | - | - | 0 | 10 (7)  (*M*=167) | 0  (*M*=203) |
| MCI | - | - | - | - | 104 (34) | 10 (7)  (*M*=167) | 0  (*M*=203) |
| Dementia | - | - | - | - | 198 (66) | 115 (85)  (*M*=167) | 99 (100)  (*M*=203) |
| *Etiologic diagnosis* (%) |  |  |  |  |  |  |  |
| Pure AD | - | - | - | - | 120 (40) | - | - |
| AD with CVD | - | - | - | - | 114 (38) | - | - |
| AD with LBD | - | - | - | - | 43 (14) | - | - |
| AD with other | - | - | - | - | 25 (8) | - | - |
| Age in years (mean [SD]) | 55.3 [5.2] | 58.2 [5.1]  (*M*=6) | 61.4 [5.1]  (*M*=15) | 64.3 [5.1]  (*M*=21) | 79.1 [5.3] | 82.6 [4.8]  (*M*=167) | 83.9 [5.0]  (*M*=199) |
| Number of females (%) | - | - | - | - | 169 (56) | - | - |
| MMSE (mean [SD]) | - | - | - | - | 21.4 [6.2] | 20.7 [6.4]  (*M*=169) | 18.7 [6.5]  (*M*=202) |
| *Study site (%)* |  |  |  |  |  |  |  |
| Forsyth | - | - | - | - | 40 (13) | - | - |
| Jackson | - | - | - | - | 112 (37) | - | - |
| Minneapolis | - | - | - | - | 64 (21) | - | - |
| Washington County | - | - | - | - | 86 (29) | - | - |
| *Race (%)* |  |  |  |  |  |  |  |
| Asian | - | - | - | - | 1 (0.3) | - | - |
| Black | - | - | - | - | 117 (39) | - | - |
| White | - | - | - | - | 184 (61) | - | - |
| NPI agitation subscale present (%) | - | - | - | - | 64 (23)  (*M*=19) | 23 (40)  (*M*=245) | 1 (6)  (*M*=286) |
| *Agitation composite total score (%)* |  |  |  |  |  |  |  |
| 0 | - | - | - | - | 164 (58)  (*M*=19) | 28 (49)  (*M*=245) | 10 (63)  (*M*=286) |
| 1 | - | - | - | - | 51 (18)  (*M*=19) | 11 (19)  (*M*=245) | 2 (13)  (*M*=286) |
| 2 | - | - | - | - | 46 (16)  (*M*=19) | 11 (19)  (*M*=245) | 4 (25)  (*M*=286) |
| 3 | - | - | - | - | 16 (6)  (*M*=19) | 5 (9)  (*M*=245) | 0  (*M*=286) |
| 4 | - | - | - | - | 6 (2)  (*M*=19) | 2 (4)  (*M*=245) | 0  (*M*=286) |
| *HRV (mean [SD])* |  |  |  |  |  |  |  |
| RMSSD* | 18.2 [11.1-31.0] (*M*=28) | 17.9 [11.9-36.7]  (*M*=33) | 17.7 [11.5-27.7]  (*M*=44) | 19.3 [11.8-29.8]  (*M*=53) | 14.5 [8.9-24.6]  (*M*=134) | - | - |
| SDNN* | 16.7 [10.4-26.6]  (*M*=28) | 16.1 [11.1-23.8]  (*M*=33) | 15.8 [10.1-24.8]  (*M*=44) | 17.4 [11.7-25.9]  (*M*=53) | 12.8 [7.9-21.0]  (*M*=134) | - | - |
| logRMSSD | 2.9 [0.7]  (*M*=28) | 2.9 [0.7] (*M*=33) | 2.9  [0.7]  (*M*=44) | 2.9 [0.7] (*M*=53) | 2.68 [0.8]  (*M*=134) | - | - |
| logSDNN | 2.8 [0.7] (*M*=28) | 2.8 [0.7]  (*M*=33) | 2.8 [0.6]  (*M*=44) | 2.9 [0.7]  (*M*=53) | 2.5 [0.8]  (*M*=134) | - | - |
| logRMMSD change^a^ (mean [SD], range) | -0.01 [0.02], -0.11-0.06 (M=4) | | | | | - | - |
| logSDNN change^a^ (mean [SD], range) | -0.01 [0.02], -0.11-0.05 (M=4) | | | | | - | - |
| *Heart rate (bpm)* |  |  |  |  |  |  |  |
| Mean [SD] | 66.1 [10.1]  (*M*=20) | 65.8, [10.4]  (*M*=14) | 65.3 [9.8]  (*M*=38) | 63.1 [10.4]  (*M*=41) | 63.9 [11.6]  (*M*=152) | - | - |
| Heart rate change^a^ (mean [SD]) | -0.13 [0.29] (M=1) | | | | | - | - |
| *Comorbidities (%)* |  |  |  |  |  |  |  |
| Diabetes | 32 (11) (*M*=4) | - | - | - | 116 (44) (*M*=40) |  |  |
| Hypertension | 108 (36) (*M*=2) | - | - | - | 222 (78) (*M*=19) |  |  |

Participants received a primary etiological diagnosis of AD at visit 5 and had or progressed to dementia by visit 7. Any missing data was reported as (*M* = number of missing data points).

*For non-normally distributed RMSSD and SDNN data, median [interquartile range] values are shown.

^a^Change in logHRV or heart rate was calculated as the slope coefficient using mixed effects models where follow up time (years) since baseline (visit 1) was the independent variable.

### **Supplementary Table 2: Characteristics of participants who were clinically diagnosed with dementia attributed solely to AD (‘pure AD’) (*n* = 120).**

| **Visit** | **1** | **2** | **3** | **4** | **5** | **6** | **7** |
| --- | --- | --- | --- | --- | --- | --- | --- |
| *Diagnostic group* (%) |  |  |  |  |  |  |  |
| Normal | - | - | - | - | 0 | 7 (13)  (M=65) | 0  (M=78) |
| MCI | - | - | - | - | 47 (39) | 6 (11)  (M=65) | 0  (M=78) |
| Dementia | - | - | - | - | 73 (61) | 42 (76)  (M=65) | 42  (M=78) |
| Age in years (mean [SD]) | 54.6 [5.0] | 57.5 [5.1] (M=2) | 60.6 [5.1] (M=7) | 63.5 [5.1] (M=8) | 78.4 [5.2] | 82.0 [5.1]  (M=65) | 83.7 [5.1]  (M=76) |
| Number of females (%) | - | - | - | - | 58 (48) | - | - |
| MMSE (mean [SD]) | - | - | - | - | 20.9 [6.5] | 20.8 [5.7]  (M=66) | 19.0 [6.3]  (M=77) |
| *Study site (%)* |  |  |  |  |  |  |  |
| Forsyth | - | - | - | - | 15 (13) | - | - |
| Jackson | - | - | - | - | 44 (37) | - | - |
| Minneapolis | - | - | - | - | 27 (23) | - | - |
| Washington County | - | - | - | - | 34 (28) | - | - |
| *Race (%)* |  |  |  |  |  |  |  |
| Asian | - | - | - | - | 1 (1) | - | - |
| Black | - | - | - | - | 47 (39) | - | - |
| White | - | - | - | - | 72 (60) | - | - |
| NPI agitation subscale present (%) | - | - | - | - | 26 (23) (M=9) | 13 (45)  (M=91) | 0  (M=110) |
| *Agitation composite total score (%)* |  |  |  |  |  |  |  |
| 0 | - | - | - | - | 64 (58) (M=9) | 12 (41)  (M=91) | 9 (90)  (M=110) |
| 1 | - | - | - | - | 24 (22) (M=9) | 6 (21)  (M=91) | 0  (M=110) |
| 2 | - | - | - | - | 15 (14)  (M=9) | 6 (21)  (M=91) | 1 (10)  (M=110) |
| 3 | - | - | - | - | 7 (6)  (M=9) | 4 (14)  (M=91) | 0  (M=110) |
| 4 | - | - | - | - | 1 (1)  (M=9) | 1 (3)  (M=91) | 0  (M=110) |
| *HRV mean [SD]* |  |  |  |  |  |  |  |
| RMSSD* | 16.9 [11.8-31.5]  (M=8) | 18.9 [12.4-28.4]  (M=15) | 16.2 [11.0-25.3]  (M=18) | 18.8 [11.4-24.6]  (M=24) | 14.4 [9.8-26.8]  (M=51) | - | - |
| SDNN* | 16.2 [9.9-27.2]  (M=8) | 17.5 [12.2-26.2]  (M=15) | 16.8 [10.0-23.1]  (M=18) | 17.0 [11.0-24.1]  (M=24) | 14.9 [8.5-22.4]  (M=51) | - | - |
| logRMSSD | 2.9 [0.7]  (M=8) | 2.9 [0.7]  (M=15) | 2.8 [0.6]  (M=18) | 2.9 [0.7]  (M=24) | 2.7 [0.8]  (M=51) | - | - |
| logSDNN | 2.8 [0.7]  (M=8) | 2.9 [0.7]  (M=15) | 2.7 [0.6]  (M=18) | 2.8 [0.6]  (M=24) | 2.6 [0.8]  (M=51) | - | - |
| logRMSSD change^a^ (mean [SD], range) | -0.01 [0.02], -0.11-0.06 (M=1) | | | | | - | - |
| logSDNN change^a^ (mean [SD], range) | -0.01 [0.02], -0.11-0.04 (M=1) | | | | | - | - |
| *Heart rate (bpm)* |  |  |  |  |  |  |  |
| Mean [SD] | 65.7 [9.3]  (M=5) | 65.5 [11.4]  (M=5) | 64.1 [10.1]  (M=10) | 61.2 [10.6]  (M=9) | 63.4 [11.7]  (M=91) | - | - |
| HR change^a^ (mean [SD]) | -0.16 [0.25] | | | | | - | - |
| *Comorbidities (%)* |  |  |  |  |  |  |  |
| Diabetes | 16 (14) (M=2) | - | - | - | 41 (41) (M=19) |  |  |
| Hypertension | 34 (29) (M=2) | - | - | - | 77 (69) (M=9) |  |  |
| *Prescribed medication(s) (%)* |  |  |  |  |  |  |  |
| Antipsychotics | 0 | 0  (M=2) | 2 (2)  (M=7) | 0  (M=8) | 4 (3) | - | - |
| SNRIs | 0 | 0  (M=2) | 0  (M=7) | 0  (M=8) | 0 | - | - |
| AChEIs | 0 | 0 (M=2) | 0 (M=7) | 0 (M=8) | 31 (26) | - | - |
| β-blockers | 7 (5.8) | 9 (8) (M=2) | 6 (5) (M=7) | 7 (6) (M=8) | 32 (27) | - | - |

Participants were diagnosed with ‘pure AD’ at visit 5 and had or progressed to dementia during the study. Any missing data was reported as (M = number of missing data points).

*For non-normally distributed RMSSD and SDNN data, median [interquartile range] values are shown.

^a^Change in logHRV or heart rate was calculated as the slope coefficient using mixed effects models where follow up time (years) since baseline (visit 1) was the independent variable.

### **Supplementary Table 3: Visit 1 (baseline) characteristics of the ‘pure AD’ subgroup by NPI agitation status**

| **Visit 1 characteristics** | **NPI agitation present (N=26)** | **NPI agitation absent (N=85)** |
| --- | --- | --- |
| Age in years (mean [SD]) | 55.0 [5.1] | 54.5 [4.9] |
| Number of females (%) | 11 (42) | 43 (51) |
| *Study site (%)* |  |  |
| Forsyth | 4 (15) | 9 (11) |
| Jackson | 7 (27) | 31 (36) |
| Minneapolis | 4 (15) | 22 (26) |
| Washington County | 11 (42) | 23 (27) |
| *Race (%)* |  |  |
| Asian | 0 | 1 (1) |
| Black | 8 (31) | 32 (38) |
| White | 18 (69) | 52 (61) |
| *HRV (mean [SD], range)* |  |  |
| RMSSD* | 15.1 [9.0-24.6], 4.4-78.2 | 17.8 [11.7-31.9], 5.0-138.4 (*M*=8) |
| SDNN* | 16.0 [7.6-25.6], 2.9-71.9 | 16.6 [10.7-29.3], 2.9-101.9 (*M*=8) |
| logRMSSD | 2.7 [0.8], 1.5-4.4 | 3.0 [0.7], 1.6-4.9 (*M*=8) |
| logSDNN | 2.6 [0.8], 1.1-4.3 | 2.9 [0.7], 1.1-4.6 (*M*=8) |
| *Heart rate (bpm)* |  |  |
| Mean [SD] | 63.0 [9.3] | 66.8 [9.4] |
| *Comorbidities (%)* |  |  |
| Diabetes | 5 (19) | 9 (11) (*M*=1) |
| Hypertension | 10 (38) | 21 (25) (*M*=2) |
| *Prescribed medication(s) (%)* |  |  |
| Antipsychotics | 0 | 0 |
| SNRIs | 0 | 0 |
| AChEIs | 0 | 0 |
| β-blockers | 2 (8) | 4 (5) |

NPI agitation subscale data, obtained at visit 5, were missing for 9 participants. Any missing values were reported (*M* = number of missing data points).

*For non-normally distributed RMSSD and SDNN data, median [interquartile range] values are shown.

^a^Change in logHRV or heart rate was calculated as the slope coefficient using mixed effects linear regression models where follow up time in years since baseline (visit 1) was the independent variable.

### **Supplementary Table 4: Characteristics of a comparison group who were cognitively normal at visits 5, 6, and 7 (*n* = 1270).**

| **Visit** | **1** | **2** | **3** | **4** | **5** | **6** | **7** |
| --- | --- | --- | --- | --- | --- | --- | --- |
| Age in years (mean [SD]) | 50.0 [4.2] | 52.9  [4.2] | 55.9 [4.2] | 58.9 [4.2] | 73.5 [4.3] | 78.4 [4.3] | 80.1 [4.3] |
| Number of females (%) | - | - | - | - | 791 (62) | - | - |
| MMSE (mean [SD]) | - | - | - | - | 28.5 [1.7] (M=1) | 28.7 [1.6] | 28.5 [1.8]  (M=11) |
| *Study site (%)* |  |  |  |  |  |  |  |
| Forsyth | - | - | - | - | 259 (20) | - | - |
| Jackson | - | - | - | - | 239 (19) | - | - |
| Minneapolis | - | - | - | - | 440 (35) | - | - |
| Washington County | - | - | - | - | 332 (26) | - | - |
| *Race (%)* |  |  |  |  |  |  |  |
| Asian | - | - | - | - | 4 (0.3) | - | - |
| Black | - | - | - | - | 263 (21) | - | - |
| White | - | - | - | - | 1002 (79) | - | - |
| Native American | - | - | - | - | 1 (0.1) | - | - |
| *HRV mean [SD]* |  |  |  |  |  |  |  |
| RMSSD* | 22.3 [14.8-33.1] (M=36) | 20.4 [13.7-30.4]  (M=44) | 19.2 [12.8-28.5]  (M=62) | 20.3 [13.2-31.1]  (M=65) | 17.7 [11.3-28.1]  (M=123) | - | - |
| SDNN* | 20.6 [13.8-30.4]  (M=36) | 19.4 [12.8-27.9]  (M=44) | 17.8 [12.3-26.3]  (M=62) | 18.7 [12.1-28.4]  (M=65) | 16.1 [10.1-25.8]  (M=123) | - | - |
| logRMSSD | 3.10 [0.64] | 3.02 [0.62] | 2.96 [0.61] | 3.02 [0.64] | 2.90 [0.75] | - | - |
| logSDNN | 3.02 [0.61] | 2.95 [0.59] | 2.89 [0.59] | 2.93 [0.63] | 2.79 [0.74] | - | - |
| logRMSSD change^a^ (mean [SD], range) | -0.01 [0.02], -0.07-0.07 | | | | | - | - |
| logSDNN change^a^ (mean [SD], range) | -0.01 [0.02], -0.07-0.07 | | | | | - | - |
| *Heart rate (bpm)* |  |  |  |  |  |  |  |
| Mean [SD] | 65.3 [8.8] | 64.3 [8.9] | 64.3 [8.7] | 61.3 [8.9] | 60.8 [9.3]  (M=34) | - | - |
| HR change^a^ (mean [SD]) | -0.19 [0.20] | | | | | - | - |

All control group participants had at least one HRV measurement. Any missing data was reported as (M= number of missing data points).

*For non-normally distributed RMSSD and SDNN data, median [interquartile range] values are shown.

^a^Change in HRV or heart rate was calculated as the slope coefficient using mixed effects models where follow up time (years) since baseline (visit 1) was the independent variable.

### **Supplementary Table 5: Associations between visit 5 logHRV (logRMSSD or logSDNN) and agitation composite scores in the ‘pure AD’ group.**

| **Linear regression models** | **Unstandardized (B) regression coefficients [95% CI]** |
| --- | --- |
| *logRMSSD* |  |
| Unadjusted | 0.01 [-0.01-0.02] |
| Adjusted 1 | 0.01 [-0.01-0.04] |
| Adjusted 2 | **0.02 [0.002-0.04]** |
| *logSDNN* |  |
| Unadjusted | 0.01 [-0.005-0.03] |
| Adjusted 1 | 0.01 [-0.003-0.03] |
| Adjusted 2 | 0.02 [-0.002-0.03] |

Statistically significant (*p* < 0.05) results are highlighted in bold. Regression coefficients are expressed for each 0.05 logHRV unit change, approximately corresponding to the observed HRV difference by 5 years of age. The relationship between logHRV and agitation was adjusted for heart rate (Adjusted 1 models) and heart rate and sociodemographic factors (visit 5 age, sex, MMSE, race-center, hypertension, and diabetes) (Adjusted 2 models).
